# miRBase: from microRNA sequences to function

**DOI:** 10.1093/nar/gky1141

**Published:** 2018-11-13

**Authors:** Ana Kozomara, Maria Birgaoanu, Sam Griffiths-Jones

**Affiliations:** School of Biological Sciences, Faculty of Biology, Medicine and Health, University of Manchester, Manchester M13 9PT, UK

## Abstract

miRBase catalogs, names and distributes microRNA gene sequences. The latest release of miRBase (v22) contains microRNA sequences from 271 organisms: 38 589 hairpin precursors and 48 860 mature microRNAs. We describe improvements to the database and website to provide more information about the quality of microRNA gene annotations, and the cellular functions of their products. We have collected 1493 small RNA deep sequencing datasets and mapped a total of 5.5 billion reads to microRNA sequences. The read mapping patterns provide strong support for the validity of between 20% and 65% of microRNA annotations in different well-studied animal genomes, and evidence for the removal of >200 sequences from the database. To improve the availability of microRNA functional information, we are disseminating Gene Ontology terms annotated against miRBase sequences. We have also used a text-mining approach to search for microRNA gene names in the full-text of open access articles. Over 500 000 sentences from 18 542 papers contain microRNA names. We score these sentences for functional information and link them with 12 519 microRNA entries. The sentences themselves, and word clouds built from them, provide effective summaries of the functional information about specific microRNAs. miRBase is publicly and freely available at http://mirbase.org/.

## INTRODUCTION

miRBase is the primary public repository and online resource for microRNA sequences and annotation (http://mirbase.org/). Established in 2002 (then called the microRNA Registry), miRBase is responsible for microRNA gene nomenclature and has been assigning gene names for novel microRNA discoveries since that time. The microRNA gene naming scheme has been discussed in previous miRBase publications (1–5), and on the miRBase blog (http://mirbase.org/blog/). The miRBase website provides a wide-range of information on published microRNAs, including their sequences, their biogenesis precursors, genome coordinates and context, literature references, deep sequencing expression data and community-driven annotation. miRBase also acts as a portal for third party information about microRNA genes and sequence, linking out to other resources such as those that include predicted and experimentally validated targets of microRNAs.

The latest release of the miRBase database (v22) contains 38 589 entries representing hairpin precursor microRNAs, from 271 organisms. This represents an increase in sequences of more than a third over the previous release. Those hairpin precursors produce a total of 48 860 different mature microRNA sequences. Vertebrate genomes contain thousands of microRNAs: for example, the human genome contains 1917 annotated hairpin precursors, and 2654 mature sequences. Well-annotated genomes of both invertebrates and plants contain hundreds of microRNAs (for example, *Drosophila melanogaster*: 258 hairpins, 469 mature sequences; *Caenorhabditis elegans*: 253 hairpins, 437 mature sequences; *Arabidopsis thaliana*: 326 hairpins, 428 mature sequences).

We discuss here recent advances and updates to the miRBase database, focusing on efforts to provide the user with more information about the quality of microRNA annotations, and the biological function of microRNA sequences.

## QUALITY OF microRNA ANNOTATIONS

The primary source of sequence data in miRBase is author submission. The overwhelming majority of microRNAs are discovered by small RNA deep sequencing approaches. As the number and depth of sequencing experiments has increased, microRNAs expressed at ever-lower abundance and with ever-more specific expression patterns have been annotated and submitted to miRBase. The number of researchers engaged in microRNA discovery has also increased, and the stringency of the criteria used by authors to annotate microRNAs has become more variable. It is therefore more and more challenging to distinguish *bona fide* microRNAs from mis-annotated fragments of other RNA species, for example. The variable quality of microRNA annotations has been discussed in the literature (see e.g. (6–10)), and concerns over annotation quality are of course of critical interest to the microRNA community. It is important to note that miRBase provides only minimal gate-keeping for quality of microRNA annotation at the point of submission. Rather, the responsibility for accuracy falls on submitting authors, and reviewers and editors of publications. miRBase aims to provide *post hoc* analyses of published microRNA sequences, such that users can assess annotation quality and select the subset of data that best matches their requirements. These analyses also trigger manual review of dubious annotations, which may lead to removal of sequences from the database. Website searches for obsolete microRNA names return the last version of the entry, and a clear reason for its removal.

Small RNA deep sequencing datasets, deposited in public databases of sequencing data such as SRA and GEO, contain enormous quantities of information about microRNA expression and biogenesis in many organisms, across developmental time, in different tissues, and in response to different external factors. The metadata deposited with RNAseq experiments does not always make it straightforward to distinguish small RNA deep sequencing datasets from whole transcriptome sets, but at the time of writing, querying the SRA database for datasets with the strategy ‘miRNA-seq’ returns 33,691 results. Around 14 000 of those datasets are from human, 5000 from mouse, 600 from *A. thaliana*, 500 from *D. melanogaster* and 300 from *C. elegans*.

Since 2010, miRBase has been collecting small RNA deep sequencing datasets, mapping reads to microRNA sequences, and showing the read mapping profiles on the website. These views have been extremely useful for examining the expression profiles of microRNAs. The canonical mechanism of microRNA processing by Drosha and Dicer proteins leads to a very specific pattern of short reads mapped across a true microRNA locus, as discussed previously (1,2,11). For example, *bona fide* microRNA genes are expected to have reads mapping to both arms of the hairpin precursor, mature microRNAs from the two arms are expected to form a duplex with 2 nt 3′ overhangs, and the 5′ end of each mature microRNA is expected to be processed with high consistency (1,11). We have separated out a subset of microRNAs that we designate as ‘high confidence’ based on support from the read data (1). A small number of sequences have mapped read patterns that are not consistent with their annotation as a microRNA, and we have been removing these entries from miRBase – 87 sequences were removed between releases 21 and 22. However, for ∼90% of microRNA annotations across the whole database, we have previously been unable to assess the confidence in their annotation due to lack of read data. Even in important animal model organisms (human, mouse, *D. melanogaster, C. elegans*), we had not collated enough read data to support or refute the validity of between 30% and 70% of microRNA annotations.

We are addressing this problem by increasing the number of datasets that we are mapping to miRBase microRNA sequences, from 426 in release 21 to 1493 in release 22. In total, these datasets contain 5.5 billion reads that map to microRNA loci, a 5-fold increase over the previous release. This increased coverage allows us to re-calibrate the criteria that we are using to call ‘high confidence’ microRNAs, and re-calculate that dataset. The criteria we are currently using are:

- The microRNA hairpin has mature microRNA sequences annotated from both arms.
- The duplex of mature microRNAs exhibits a 3′ overhang of 0–4 nt.
- Each mature microRNA has ≥20 overlapping reads.
- ≥50% of the reads mapping to each mature microRNA have the same 5′ end.

**Figure 1.**
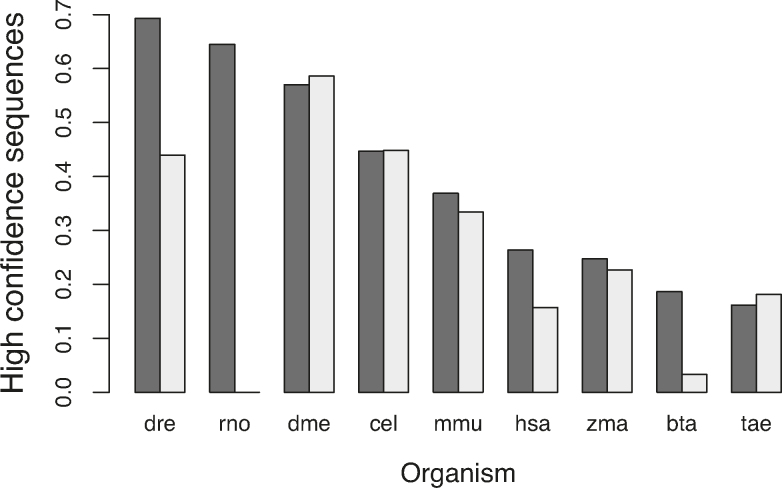
The proportion of microRNA sequences annotated as high confidence in release 22 (black) and release 21 (grey). All species with 30 or more small RNA deep sequencing datasets in miRBase 22 are shown. Species abbreviations: dre: *Danio rerio*; rno: *Rattus norvegicus*; dme: *Drosophila melanogaster*; cel: *Caenorhabditis elegans*; mmu: *Mus musculus*, zma: *Zea mays*; hsa: *Homo sapiens*; bta: *Bos taurus*; tae: *Triticum aestivum*.

Despite the increased stringency of these rules (we previously required only 10 reads per arm), the proportion of microRNA annotations that are classified as ‘high confidence’ in many well-studied organisms has increased (see Figure 1). For example, 26% of human microRNA annotations are classified as ‘high confidence’ in miRBase 22, compared with only 16% in miRBase 21.

As previously, it is important to note that the main reason that microRNAs are not classified as ‘high confidence’ is lack of data. For example, 1225 human microRNAs (64%) do not have ≥20 reads associated with each arm in the datasets that we have collected. Lack of data is not sufficient to assert that these microRNAs are not valid annotations. However, some microRNA annotations have lots of mapped reads, but the pattern of those reads does not support the processing of a microRNA by Drosha and Dicer. We have therefore introduced a ‘low confidence’ classification, currently defined using the following rules:

- More than 100 reads map across the microRNA hairpin locus.
- <30% of reads mapping to the more abundant arm of the hairpin have the same 5′ end.

A total of 245 annotations are currently flagged as ‘low confidence’ in the database, including 17 human entries. We are in the process of manually reviewing each one, and removing from miRBase where appropriate.

We previously introduced a simple voting feature, allowing miRBase users to vote for or against the validity of any given microRNA annotation (1). This feature has been well-used: over 13 000 votes have been cast across over 4000 different microRNA sequences from 199 species. For example, in human, 7644 votes are registered against 1195 of the 1917 sequences (62%). In total, 725 microRNA sequences have an overall negative voting score. 208 of those negative scores are human sequences, and 19 of those have a score of –5 or less. We have already used these data to highlight sequences for review, and as a result have removed members of the mir-566, mir-1273, mir-4419 and mir-6723 families from the database. 10 additional families highlighted in this way are flagged for future removal. These growing data supplement our automated confidence assignments, and the visual representations of read data, to allow users to quickly judge the quality of any microRNA annotation in miRBase.

## BIOLOGICAL FUNCTIONS OF microRNAs

The spatial and temporal expression of microRNAs inferred from deep sequencing datasets gives clues to their function. For example, mouse mir-1 family members have well-described roles in muscle development (12,13), and the read data shown for mmu-mir-1a-1 in miRBase clearly shows its high expression in both cardiac and skeletal muscle.

However, extensive functional annotation of microRNAs has generally been lacking in miRBase. For example, the scientific articles cited for each microRNA entry generally refer to the microRNA discovery, rather than to function. Information about the functional roles of microRNAs is available elsewhere in a variety of forms. For example, there are a number of well-established and well-used databases of both predicted and validated microRNA targets. For example, TargetScan (14), DIANA-microT (15) and miRDB (16) are amongst many resources that provide web interfaces to search and view the predicted target sites of most microRNAs. These resources often update their predictions in line with new microRNA releases through miRBase. The TarBase (17) and miRTarBase (18) databases curate and provide lists of microRNA targets that have some experimental support. miRBase does not curate or collate predicted or validated target sets, but rather links from entries to external target resources. We have worked to improve and increase these links from miRBase. Over a fifth of mature microRNAs (10 609/48 860) in miRBase have links to target predictions, and 4154 (8.5%) link out to validated target sets. Those proportions are much higher for the best-studied and most viewed organisms – for example 2578 and 2599 of the 2654 human mature sequences in miRBase have links to predicted and validated targets respectively.

### Gene Ontology

An enormous quantity of functional information is locked up in the scientific literature. As of Sept 2018, over 80 000 articles in PubMed contain the term ‘microRNA’ or ‘miRNA’ in the title, keyword or abstract, including 13 726 published in 2017. Extracting biological information from these papers in any kind of structured way is difficult and time-consuming.

The Gene Ontology (GO) provides a controlled and flexible structure by which functional information can be attached to genes (19,20). The widespread assignment of GO terms to protein-coding genes has been one of the success stories of bioinformatics and genomics, driven by the adoption of GO as a standard amongst model organism and genome browser resources, and enormous annotation efforts by the GO Consortium (20). The manual curation of terms for a given gene involves an expert biocurator reading scientific papers, extracting and interpreting statements related to the function of the gene of interest, and attaching that information to the gene in the form of terms from the structured ontology. However, until recently, there had been little coordinated effort to annotate GO terms to non-protein-coding genes. These data have not therefore been widely available in bioinformatics databases or resources.

Recently, the UCL Functional Gene Annotation team has embarked on an effort to annotate microRNAs with GO terms (21,22). In total, the team have annotated over 500 mature microRNAs from human, mouse and rat with nearly 5000 GO terms, over 3000 of which are linked with human microRNAs (22). They have also annotated information on ∼2500 experimentally-validated targets of mature microRNAs. These datasets of microRNA GO annotations, together with annotations that are starting to be deposited by resources such as MGI (23) and RNAcentral (24), are made available through the QuickGO resource at EBI (25). RNA genes in QuickGO are identified using sequence identifiers from the RNAcentral database. Using the EBI webservices and an RNAcentral-provided mapping from miRBase accessions to RNAcentral identifiers, we are able to extract the set of GO annotations associated with any miRBase entry, for display on the miRBase entry pages. Table 1 shows an example set of annotations for the hsa-miR-499a-5p mature microRNA. The annotations show that the microRNA directly regulates the genes SOX6 and KCNN3, and has a role in cardiac muscle cell differentiation (26,27). These high-value manually-annotated datasets complement the more extensive automated target prediction and validation datasets discussed above.

**Table 1.** Gene Ontology annotations for hsa-miR-499a-5p

Qualifier	GO term	Evidence	Notes	Reference
*involved_in*	GO:0014883 transition between fast and slow fiber	ECO:0000250 sequence similarity evidence used in manual assertion	*occurs_in* UBERON:0001389 soleus muscle	GO_REF:0000024 Manual transfer of experimentally-verified manual GO annotation data to orthologs by curator judgment of sequence similarity
*involved_in*	GO:0035195 gene silencing by miRNA	ECO:0000314 direct assay evidence used in manual assertion	*regulates_expression_of* ENSG00000110693 SOX6 *occurs_in* CL:0010021 cardiac myoblast *regulates* GO:0055007 cardiac muscle cell differentiation	PMID:20081117
*involved_in*	GO:0035195 gene silencing by miRNA	ECO:0000314 direct assay evidence used in manual assertion	*regulates_expression_of* ENSG00000143603 KCNN3	PMID:23499625
*involved_in*	GO:2000727 positive regulation of cardiac muscle cell differentiation	ECO:0000314 direct assay evidence used in manual assertion	-	PMID:20081117
*involved_in*	GO:2000818 negative regulation of myoblast proliferation	ECO:0000314 direct assay evidence used in manual assertion	*results_in_division_of* CL:0010021 cardiac myoblast	PMID:20081117
*enables*	GO:1903231 mRNA binding involved in posttranscriptional gene silencing	ECO:0000314 direct assay evidence used in manual assertion	*has_direct_input* ENSG00000110693 SOX6	PMID:20081117
*enables*	GO:1903231 mRNA binding involved in posttranscriptional gene silencing	ECO:0000314 direct assay evidence used in manual assertion	*has_direct_input* ENSG00000143603 KCNN3	PMID:23499625

### Mining the scientific literature for microRNA function

The manual curation of information from the scientific literature provides gold-standard quality functional annotation (22). However, this work is obviously enormously labour-intensive, and as a result, less than 10% of mature miRNA sequences in human, mouse and rat currently have this level of information. It is therefore useful to supplement such efforts with automated text-mining approaches. Automated extraction of functional annotation is difficult. Recognizing gene names, or even species names, in full-text articles is surprisingly challenging (28–30). For example, many gene names are common English words, a problem that is particularly acute in species such as *D. melanogaster*. However, with just a handful of exceptions, microRNA gene names, as assigned by miRBase, have been standardized in format in the scientific literature since 2002. Almost all microRNA gene names are therefore unambiguous and straightforward to recognize automatically in full-text searches.

**Figure 2.**
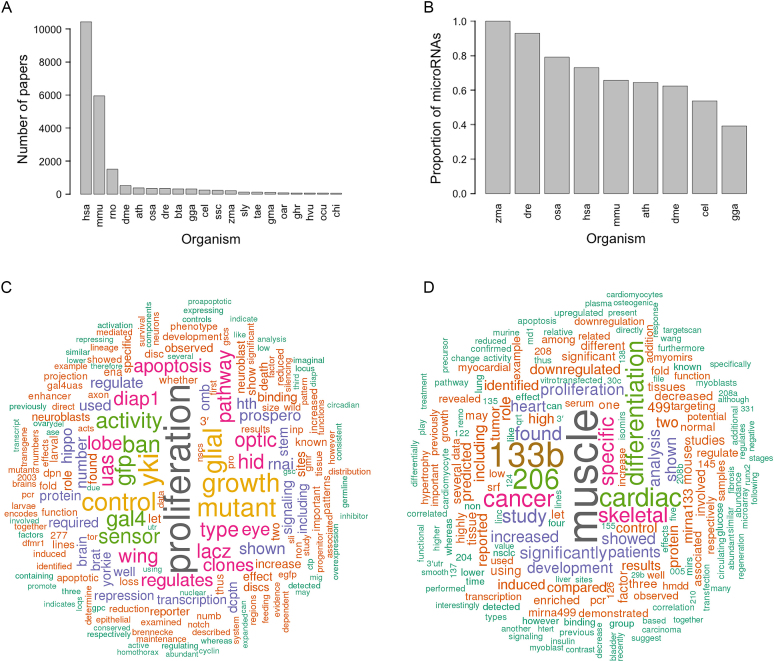
MicroRNA functional information mined from open access papers. (**A**) The top 20 species according to the number of open access papers associated with their microRNAs. (**B**) The proportion of microRNAs from selected model organisms that have papers and sentences associated. (**C**) Word cloud for *Drosophila* melanogaster bantam microRNA. (**D**) Word cloud for hsa-mir-133a-2. Species abbreviations: hsa: *Homo sapiens*, mmu: *Mus musculus*, rno: *Rattus norvegicus*; dme: *Drosophila melanogaster*; ath: *Arabidopsis thaliana*; osa: *Oryza sativa*; dre: *Danio rerio*; bta: *Bos taurus*; gga: *Gallus gallus*; cel: *Caenorhabditis elegans*; ssc: *Sus scrofa*; zma: *Zea mays*; sly: *Solanum lycopersicum*; tae: *Triticum aestivum*; gma: *Glycine max*; oar: *Ovis aries*; ghr: *Gossypium hirsutum*; hvu: *Hordeum vulgare*; ocu: *Oryctolagus cuniculus*; chi: *Capra hircus*.

We have adopted the following procedure to mine the scientific literature for functional information about microRNAs. We have downloaded the PubMed Central open access corpus of literature (ftp://ftp.ncbi.nlm.nih.gov/pub/pmc/oa_bulk/; 21 July 2018). Initially, we are working with the for-commercial-use subset, containing 1 252 865 full-text articles, but will be extending the analysis to include the non-commercial-use dataset. The Organisms database (https://organisms.jensenlab.org/; 21 July 2018) was used to associate each full-text article with a list of organisms (30). Text from the abstract, introduction, results and discussion sections was extracted from the XML file for each publication and split into sentences using the python library ntlk.tokenize (v.3.3). Each sentence was then searched for microRNA gene name mentions using regular expressions (including ‘miR-#’, ‘miRNA-#’, ‘microRNA-#‘ and the exceptional microRNA names let-7, lin-4, bantam, etc). 18 542 of the articles analysed (1.48%) contain a microRNA gene name. The top 20 species with which articles mentioning microRNA gene names have been associated are shown in Figure 2A.

To separate real sentences from extracts of tables and references, sentences containing >25 different microRNA gene names or >200 words in total were eliminated. The remaining sentences containing microRNA gene names were then associated with the species identified for that paper in the Organisms database. There are two obvious sources of mis-assignment of papers to microRNA genes in a specific organism:

- Many papers are associated with more than one species. Sentences not containing the name of the species for a given microRNA, but containing names or abbreviations of names of other species, were therefore excluded. However, if a sentence contains no species name or abbreviation, we cannot tell whether the microRNA gene name refers to one or all of the species linked with the paper. In these cases, we currently assign the paper and sentences to all associated species.
- Many microRNA mentions in papers are at the gene family level. Where we cannot tell which family member a sentence refers to, we assign the paper and sentence to all family members in the relevant organism. For example, the term ‘let-7’ in a paper associated with the species *Homo sapiens* causes the sentence to be associated with all 11 human let-7 family members.

We have used a simple scoring scheme to rank microRNA name-containing sentences according to the inclusion of possible functional terms. Keywords related to microRNA function (e.g. ‘expression’, ‘target’, ‘regulate’, ‘inhibit’) contribute a positive score, while negative scores are triggered by keywords linked to measurements, calculations or experimental approaches. The term list will be constantly refined to generate the most useful sentence ranking. The papers associated with a microRNA are ranked according to the sum of the sentence scores.

After filtering, a total of 554 287 sentences have been associated with 12 519 microRNAs. The proportion of microRNA genes that have papers and sentences associated varies across the well-studied model organisms (see Figure 2B). For example, 73% of human microRNA genes (1401/1917) have associated papers and sentences. The gene hsa-mir-21 has the largest number of linked papers: 1559, with 16 584 sentences mentioning the microRNA. Titles of the top 10 articles by summed sentence score for hsa-mir-21 are shown in Table 2.

**Table 2.** The top 10 ranked articles for hsa-mir-21, the number of sentences associated with the microRNA name, and other human microRNAs associated with the paper

Pubmed ID	Article title	Sentences	Other human miRNAs
22685542	MicroRNA-21 governs TORC1 activation in renal cancer cell proliferation and invasion	224	–
26975392	Relevance of miR-21 in regulation of tumor suppressor gene PTEN in human cervical cancer cells	145	let-7a-1, let-7a-2, let-7a-3, mir-214
20113523	MicroRNA-21 inhibitor sensitizes human glioblastoma cells U251 (PTEN-mutant) and LN229 (PTEN-wild type) to taxol	116	mir-221, mir-222, mir-328, mir-451a, mir-451b
25058005	Alteration in Mir-21/PTEN expression modulates gefitinib resistance in non-small cell lung cancer	109	mir-181b-1, mir-181b-2, mir-214,
26160841	Inhibition of miR-21 restores RANKL/OPG ratio in multiple myeloma-derived bone marrow stromal cells and impairs the resorbing activity of mature osteoclasts	114	mir-29b-1, mir-29b-2, mir-34a, mir-221, mir-222, mir-9718
22931209	miRNA-21 is developmentally regulated in mouse brain and is co-expressed with SOX2 in glioma	119	mir-16-1, mir-16-2, mir-29a, mir-29b-1, mir-29b-2
23717555	4-HNE increases intracellular ADMA levels in cultured HUVECs: evidence for miR-21-dependent mechanisms	97	-
21544242	MiR-21 induced angiogenesis through AKT and ERK activation and HIF-1α expression	81	let-7^#^, mir-27b, mir-130a, mir-126, mir-296, mir-378^#^
23082189	Mechanical stretch modulates microRNA 21 expression, participating in proliferation and apoptosis in cultured human aortic smooth muscle cells	93	mir-19a, mir-23b, mir-26a-1, mir-26a-2
23991015	MicroRNA-21 in pancreatic ductal adenocarcinoma tumor-associated fibroblasts promotes metastasis	113	mir-122

#All family members are associated with the article.

We have built word cloud representations of the complete set of sentences associated with each microRNA, using the R packages tm and wordcloud. The tm package is used to remove English language stop words, and common scientific terms that obscure the more useful functional information are also curated and removed (e.g. ‘expressed’, ‘cell’, ‘sequence’, ‘gene’). Two example word clouds are shown in Figure 2. These images give an immediate visual summary of the keywords associated with a given microRNA. For example, the *D. melanogaster* bantam word cloud (Figure 2C) is built from 712 sentences from 108 open access papers. The word cloud highlights a number of terms related to its role in regulating the neural stem cell proliferation, its key apoptotic target gene hid, and its links with the hippo pathway (31–33). The word cloud built from 1750 sentences from 381 papers linked to the microRNA hsa-mir-133a-2 (Figure 2D) clearly highlights the known roles of mir-133 genes in both cardiac and skeletal muscle development (34,35). It also shows that mir-133 co-occurs in sentences with other microRNAs, including mir-206 and mir-499, both of which are known to regulate cardiac muscle differentiation (35,36).

A new interface to the papers and sentences associated with each microRNA has been built for the miRBase website. These pages and the associated word clouds are prominently linked from each microRNA entry page. Currently, we show word clouds only for microRNAs that have 10 or more associated sentences. We hope that this interface provides an efficient and useful first view of the functional roles of a microRNA, and an easy way to identify and access the most informative scientific articles about microRNA function for deeper exploration. For example, we envisage that this interface will be useful for fast identification of interesting microRNAs from a list of candidates resulting from a differential expression experiment. We also hope that it will prove a useful tool for expert biocurators who produce gold-standard functional annotation datasets (see above), helping them to quickly access the most useful articles.

## FUTURE DEVELOPMENTS

The three datatypes described here—deep sequencing data, GO annotations, and open access articles—are all expected to grow rapidly. Despite our focus on increasing the coverage of small RNA deep sequencing datasets represented in miRBase, we have only collected around 5% of the available data. We will therefore prioritise identifying and incorporating those datasets that will help us to assess the validity of the widest set of miRBase entries. For the GO annotations, the use of QuickGO webservices to build the available data into miRBase web pages means that new annotations and edits will be visible in miRBase as they are made. We will also update our analysis of open access papers, including the non-commercial-use dataset, and new papers as they are published.

We are particularly keen to further develop the text-mining approaches to extract functional information. The sentence-scoring scheme will be continuously adapted and improved to rank sentences and papers in the most useful way. However, no automated scoring function will match a biologist's ability to assess the usefulness of a given sentence or paper. We are therefore considering integrating a community feedback feature into the sentence scoring. For example, users of miRBase could vote sentences up and down according to the quality of biological information they provide.

## DATA AVAILABILITY

All miRBase data are publicly and freely available under the Creative Commons Zero license. Data are available for bulk download from ftp://mirbase.org/ and on the web at http://mirbase.org/. Feedback on any aspect of the miRBase database, and discussion of novel microRNA sequence names, are welcome by email to mirbase@manchester.ac.uk.
